# Aging and DNA methylation

**DOI:** 10.1186/s12915-015-0118-4

**Published:** 2015-01-31

**Authors:** Marc Jung, Gerd P Pfeifer

**Affiliations:** Beckman Research Institute, Duarte, CA 91010 USA; Van Andel Research Institute, Grand Rapids, MI 49525 USA

## Abstract

In this Opinion article, we summarize how changes in DNA methylation occur during aging in mammals and discuss examples of how such events may contribute to the aging process. We explore mechanisms that could facilitate DNA methylation changes in a site-specific manner and highlight a model in which region-specific DNA hypermethylation during aging is facilitated in a competitive manner by destabilization of the Polycomb repressive complex.

## Introduction

Aging can be described as a slow, time-dependent decline of a set of multiple biological functions. In some biological pathways, functional decline can be defined in a mono-causal way, such as the decline of resting metabolism, whereas in other pathways the scope of the decline is rather broad and elusive, such as that for reduced stability of epigenetic patterns. Although epigenetic patterns change dramatically during development, these early events are biologically programmed and necessary, whereas alterations of the epigenome in adult somatic tissue may reflect aging-associated deleterious events. Efforts have been made to encapsulate processes of aging into well-defined categories. These range from accumulation of genomic damage that leads to chromosomal instability and telomere shortening, reactive oxygen species-induced damage to mitochondrial functions and reduced energy production, stem cell depletion, accumulation of damaged proteins via loss of proteostasis, processes leading to senescence and changes in intercellular communications, age-related effects of the insulin and IGF-1 signaling pathways and, lastly, alterations of the epigenome. Many of these altered pathways are thought to be the prime components of age-related diseases including cancer, neurodegenerative diseases, atherosclerosis and inflammation. A detailed description of these categories can be found in the review by López-Otín *et al*. [1], which defined these categories as the ‘hallmarks’ of aging. It is expected that there is extensive crosstalk between categories and because epigenetic changes are often regulatory events and can lead to altered gene expression, they can impact other hallmarks of aging, such as by silencing of DNA repair genes or anti-inflammatory genes.

Here, we would like to focus on the epigenetic contributions to aging. More specifically, we will discuss the relationship between aging and changes in DNA methylation. DNA methylation patterns are shaped by two opposing processes of adding and removing a methyl group at position five of cytosine in DNA. The first functions of DNA methylation to be discovered were related to gene regulation and cell differentiation [2]. Selective maintenance of DNA methylation at specific loci is essential for controlling differential expression of the paternal and maternal alleles in mammals [3], known as genomic imprinting. In particular, X-chromosome inactivation is an often-cited example where DNA methylation is required for long-term silencing of a locus [4]. After the developmental phase, the genome of somatic cells will consist of roughly 1% methylated DNA cytosines, mostly affecting CpG dinucleotides [5]. While there exists great variability between the established patterns of DNA methylation, there is a consistent landmark in the form of CpG islands, which are unmethylated GC-rich regions with high densities of CpGs and often correlated with promoter regions [6]. After DNA methylation patterns have been established during embryogenesis, key questions in the field are how regulatory mechanisms define and maintain them in specific tissues, how the environment can facilitate changes in methylation patterns during a lifespan and what the impacts of these changes are.

## Predictability of DNA methylation during aging

The earliest studies, which related DNA methylation changes to the aging process, were investigating different organs and life stages of humpback salmon [7] and found that 5-methylcytosine (5mC) levels during ontogenesis were significantly decreasing. Attesting to the evolutionary importance of DNA methylation, these results could be later extended to mammals [8]. The highest amount of 5mC was observed in embryos and then followed a seemingly gradual decrease. In rodents it was shown that the decrease of 5mC was inversely related to lifespan [9]. A link between global DNA hypomethylation and senescence could be further strengthened by experiments with fibroblast cultures [10] and by the observation that the DNA methylation inhibitor 5-aza-2′-deoxycytidine substantially shortened the lifespan of cells [11]. A comparison between the DNA methylomes of CD4^+^ T cells of newborns and centenarians, using whole genome bisulfite sequencing, verified the overall hypomethylation as a function of age. Newborn CpG methylation was more homogeneous compared to the centenarian methylomes, indicating a scattered demethylation over a lifetime [12]. However, these data were based on cross-sectional studies (groups of different individuals of different age). Longitudinal studies (same individuals at two or more time points) are rare. One such longitudinal study examining global levels of methylation in white blood cells found roughly the same number of participants with a decrease in methylation as with an increase [13]. Such substantial intra-individual change in opposite directions would likely be missed by age-specific cross-sectional analysis.

Global DNA hypomethylation also does not mean that individual sites become hypomethylated during aging. The gene encoding the estrogen receptor (ER) was the first gene shown to become hypermethylated in colon with increasing age [14]. These findings led to genomic studies addressing the question of whether at a genome-wide level the methylation patterns of a specific set of genes could be used to predict the age of individuals. The continuous accumulation of genome methylation data in the form of Infinium HumanMethylation BeadChip assays (Illumina) enabled a systematic evaluation of this question. In one case, Florath *et al*. [15] could identify from 962 whole blood DNA samples a set of 162 CpG sites that were predictive for the age of the donors. While the general concept of an epigenetic clock was already established [16], a breakthrough in the data analysis was the work of Horvarth [17], who used not only one specific tissue but a collection of different tissues from 8,000 samples. Indeed, he was able to identify a tissue-independent set of 393 CpG sites that could predict the age of the donors with a correlation of 0.96 and an error of 3.6 years remarkably well. More recently, at least for blood DNA, the number of CpG sites needed for age prediction could be reduced to just three [18], establishing that only a very few selected CpG sites are sufficient for reliable age prediction. Thus, there is substantial evidence for an ‘epigenetic clock’ model.

On a functional level, we have to note that the common canon that increasing DNA methylation in a promoter leads to decreased gene expression and vice versa cannot be generalized. For example, in human peripheral blood mononuclear cells (PBMCs), the overall correlation of gene expression and DNA methylation changes was weak, arguing against a model where age-related DNA methylation changes would generally exert a biological role via gene expression changes [19].

Another challenge when studying aging via DNA methylation changes is confounding effects, which affect DNA methylation over a long time period as well. As such it is known that type 2 diabetes and associated obesity have an effect on the methylome [20,21]. The quality of air we breathe could have an impact on the overall methylation pattern [22] as well as more specific toxic exposures over a longer time period [23]. Also life style decisions, as diverse as exercise and aspirin usage [24] or smoking [25], can have significant effects. Even physical environmental parameters have been shown to have an effect on DNA methylation [26]. Although it is not unreasonable to expect associations between environmental factors or exposures and DNA methylation patterns, reproducing such effects in independent studies will be required before any firm conclusions that they are confounders can be reached. Often, information about the lifestyle, eating habits, air quality, body mass index, recorded diseases, and living conditions is lacking from age-related DNA methylation studies. Apart from the ‘clock-specific’ age signatures, without this information it is impossible to attribute changes in DNA methylation over time to intrinsic age-specific effects.

## Mechanisms of DNA methylation changes during aging

When looking for functional overlaps with other genomic features, the DNA hypermethylation of Polycomb group protein (PcG) target genes is emerging from both aging- and cancer-related studies as a common theme. Polycomb group proteins form complexes that associate with DNA and chromatin and normally function as repressors of genes involved in embryonic development and cell-fate decisions [27-30]. The function of the Polycomb repressive complex 2 (PRC2) is to tri-methylate lysine 27 on histone H3 (to create H3K27me3), which is a histone modification maintained during cell division [31]. PRC2 mediates transcriptional silencing via its H3K27me3 mark, which is either subsequently or independently bound by a subset of Polycomb repressor complex 1 (PRC1) protein complexes, leading to further chromatin compaction and acting as a stable silencing mechanism [32,33]. Polycomb group protein target genes have been shown to be prone to hypermethylation in diverse types of cancer [34-39] and a recent study in breast cancer showed the close relationship to age-related DNA methylation changes [40]. It is still not exactly clear how Polycomb complexes are targeted to specific genomic regions but growing evidence supports the theory that DNA methylation patterns are involved in regulating PcG accessibility and vice versa. Further evidence for Polycomb targeting to CpG islands comes from a Dnmt3a/b^−/−^ mouse embryonic stem cell model, suggesting that a high density of unmethylated CpG dinucleotides is sufficient for PcG recruitment [41].

One study, investigating genome-wide DNA methylation changes by comparing C57BL/6 mice at 3 months of age to mice at 35 months of age, found that Polycomb target genes and various tumor suppressor genes were enriched among the age-dependently hypermethylated genes [42]. There is substantial crosstalk between DNA methylation and the Polycomb mark. Recent studies showed that increased H3K27me3 occupancy occurred at regions of the genome that are normally highly DNA-methylated and then become demethylated after inhibition of DNA methylation by 5-aza-2′-deoxycytidine (5-aza-dC) [43]. One specific mechanism by which the Polycomb complexes recognize unmethylated DNA regions is via the protein KDM2B, which recruits PRC1 and PRC2 [44].

**Figure 1:**
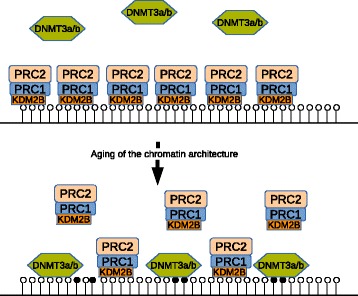
***De novo*** **methylation by competition.** Unmethylated CpG-rich DNA regions, termed CpG islands, are recognized and bound by KDM2B, which recruits PRC1, which in turn results in PRC2 binding and H3K27me3 formation. Our theory is that this complex degrades with age, allowing gradual access of DNA to the *de novo* methyltransferases DNMT3A and DNMT3B and leading to partial DNA methylation in older individuals. A subset of the Polycomb-marked genes will simultaneously carry an active chromatin mark, H3K4me3, and are referred to as bivalent genes. A similar process may be operative, but for bivalent genes both the H3K27me3 modification and the H3K4me3 mark need to be lost in order for DNA methylation to occur. White circles illustrate unmethylated CpG sites and black circles show methylated CpG sites.

How can unmethylated regions bound by the Polycomb complex become methylated in an age-dependent manner? One theory of abnormal methylation during aging involves the recruitment of *de novo* DNA methyltransferases by the Polycomb complex [45]. We propose an alternative theory where the targeting of the PcG machinery to unmethylated CpG-rich target sequences erodes with age, allowing DNA methylation at PcG target genes to increase slowly over time. This would suggest a competitive model where unmethylated DNA regions are protected by PRC1 and PRC2 complexes and as these get continuously degraded during aging, becoming more and more accessible for the DNA methyltransferases DNMT3A and/or DNMT3B, which facilitate *de novo* DNA methylation (model in Figure 1). The replacement of the Polycomb mark with DNA methylation also occurs in cancer and has been referred to as Polycomb switching [46]. The main outcome of this switch is a loss of plasticity inasmuch as DNA methylation is considered a much more stable modification associated with inactive chromatin.

## Age-specific DNA methylation changes and age-related diseases

While there are undeniable facts that DNA methylation patterns change over time, not many of those have been thought to play a role in age-related diseases. The number one age-related disease is cancer and indeed one of the best predictors of tumors is the age of patients [47]. In most cancer types, global DNA hypomethylation can be observed [48,49], which is a potential causal factor in reducing genome stability and increasing chromosomal aberrations. There is evidence that this can be at least partially attributed to the specific hypomethylation of repetitive regions and the resulting reactivation of retrotransposons [50]. But not only global DNA hypomethylation can contribute to early events in neoplastic cell transformation. For some genes, site-specific hypermethylation of promoter regions are found in both aging and tumorigenesis, which makes them *bona fide* candidates for increased cancer susceptibility. Examples include insulin-like growth factor-II (*IGFII*), hypermethylated in cancer 1 (*HIC1*), caspase-8 (*CASP8*), glutathione S-transferase pi (*GSTP1*), suppressor of cytokine signaling 1 (*SOCS1*), RAS association domain family 1A (*RASSF1A*), p16/*CDKN2A*, adenomatosis polyposis coli (*APC*) and the aforementioned estrogen receptor 1 (*ESR1*) [51-58]. DNA hypermethylation of these genes was identified in not one but several tumor types. Also DNA hypermethylation in *HOX* genes or in certain genes encoding lineage-specific transcription factors identified in small cell lung cancer could represent an epigenetic component of an age-related process, pushing cell fate towards transformation [39].

Not only cancer but also inflammatory diseases show an age-related increase of DNA methylation. Inflammation is often associated with DNA hypermethylation of specific genes as initially reported for ulcerative colitis [59]. DNA hypermethylation in inflamed tissues is also strongly targeted to genes recognized by the Polycomb complex [38]. As inflammatory processes increase with age, it is expected that DNA methylation changes would be accelerated in affected tissues. A more causal connection between DNA hypomethylation and atherosclerosis could be established by the use of atherosclerosis-prone *Apoe*^*−/−*^ mice [60]. Also, promoter-related hypomethylation linked to transcriptional upregulation of key enzymes was linked to atherosclerosis. Furthermore, the gradual decrease of methylation in promoter fragments of the tumor necrosis factor alpha (TNFα) gene, which is important for inflammatory reactions, could be a main factor in the onset of chronic inflammatory conditions with older age [61]. Another age-associated disease is type 2 diabetes. And indeed, a change of DNA methylation patterns can lead to perturbed glucagon and insulin secretion [62]. Finally, neurodegenerative diseases like Alzheimer’s disease, which is correlated with age, show support for an underlying mechanism based on reduced levels of DNA methylation. The amyloid precursor protein (*APP*) gene in the Alzheimer’s disease brain showed gradual hypomethylation in the promoter [63]. Age-dependent changes of methylation for the presenilin 1 (*PS1*) gene, which is essential for the formation of the γ-secretase complex, also imply gene expression changes [64,65]. In summary, DNA methylation changes are becoming a factor increasingly linked to our understanding of age-related pathologies.

## Age-specific DNA methylation changes and metabolism

Models that try to explain the global reduction of DNA methylation during a lifetime are inevitably bound to include metabolic changes. Resting metabolic rates have been shown to decrease during aging [66] and those processes involved in DNA methylation and de-methylation are embedded in the one-carbon metabolism, which is required for methionine biosynthesis and cellular methylation reactions [67,68]. The methyl groups needed for DNA methylation are added from S-adenosylmethionine (SAM), the primary universal donor of methyl groups in mammals derived from methionine [69]. One mechanism explaining a potential decrease of 5-methylcytosine seems to work through specific hypomethylation caused by increased levels of homocysteine and S-adenosylhomocysteine (SAH), which leads to inhibition of cellular methylation reactions [70]. In general, the one-carbon metabolic pathway can be impaired by genetic or external variation, more specifically by nutritional habits. And as such, it is currently assumed that regular intake of nutrients involved in the metabolism of the methyl group, like folic acid or vitamin B12, can slow down the gradual hypomethylation observed during the aging process [71,72]. However, the relationship between folate status and DNA methylation levels is complex and is likely influenced by folate availability [73].

Not only one-carbon metabolism-related processes lead to global hypomethylation: global hypomethylation can also occur via a peroxisome proliferator-induced mechanism [74]. Also, there are some hints based on cell culture experiments that altered DNA methylation events are correlated to Sirt1 expression levels, which may play a pivotal role in the beneficial effects of dietary restriction, which itself is believed to extend lifespan [75]. The enzymes, which transfer a methyl group from SAM to DNA producing 5-methylcytosine, are the family of DNA methyltransferases (DNMTs) that include DNMT1, DNMT3A, DNM3B and DNMT3L [76]. DNMT1 plays an important role in maintaining genomic methylation patterns [77]. How is DNMT1 linked to aging? The activity of DNMT1 seems to decrease significantly during aging [78,79], and this is seen as a viable model to explain the global decrease of DNA methylation observed during aging. Furthermore, the study of a *Dnmt1*^*+/−*^ mouse model observed a lower DNA methylation level, which led to impaired learning and memory functions in an age-dependent manner [80]. The reduction in DNMT1 can in theory be explained by an age-related decrease of growth hormone as there is evidence that the expression of Dmnt1 and Dmnt3a is influenced by growth hormone, establishing a link between DNA methylation and the IGF-1/FOXO pathway, which plays a profound role in the aging process and was first discovered in *Caenorhabditis elegans* [81].

Another potential mechanism for DNA demethylation during aging is by enzymatic DNA demethylation catalyzed by the 5mC dioxygenases Ten-eleven translocation 1, 2, and 3 (TET1/2/3) [82,83]. But while the function of TET proteins has been linked to reprogramming in early development and to cancer, their role in aging has remained elusive [84]. In summary, metabolic processes, orchestrating the homeostasis between SAM, SAH and the key DNMT enzymes and potentially TET proteins, may have a long-term impact on the specific rate of aging, but more definitive causal connections still remain to be established.

## Tissue specificity of age-related DNA methylation changes

While there is evidence for a tissue-independent, age-related change in DNA methylation [85], which might become a useful tool for determining an individual’s age, for example at crime scenes, the rate of the increase is generally varied depending on the tissue [86]. One hypothesis to explain this is that the level of exposure to environmental agents of a given tissue type, with a high exposure, for example, in skin and colon, reflects a higher correlation with DNA methylation changes over time. A recent study by Day *et al*. [87] identified age-related CpG DNA methylation changes in human blood, brain, kidney and skeletal muscle tissue. Interestingly, hypomethylated CpG sites were more strongly related to tissue-specific, age-related changes compared to hypermethylated CpG sites. Skeletal muscle showed the strongest links to tissue-specifically expressed genes and proximity to CTCF binding sites. Another study, based on blood DNA, also implied no predictive power for other tissues [88].

## Stem cell aging

When analyzing tissues composed of multiple cell types, it is important to take into account stem cell sub-populations, which might represent a signature specific for stem cell functions. Accumulated abnormal DNA methylation during a lifespan is believed to have an impact on the behavior and functionality of stem cells [89]. It was shown that DNA methylation and inactivation of myeloerythroid genes protects hematopoietic stem cells (HSCs) from premature differentiation, suggesting a link to tissue homeostasis [90]. Deterioration of stem cell function is one of the hallmarks of aging that is thought to lead to several types of age-associated pathologies, including various degenerative diseases and dysfunction and malignancies in the hematopoietic system. While the mechanisms of hematopoietic stem cell decline with age are complex, epigenetic alterations are likely involved. A recent study analyzing DNA methylation changes in HSCs from old versus young mice identified small but significant changes in cytosine methylation patterns [91]. These authors found that alterations of DNA methylation occurred at genomic regions associated with hematopoietic lineage potential and selectively targeted genes expressed in downstream progenitor and effector cells. The observed hypermethylation events have the potential to restrict access to chromatin regions of lineage-regulating factors and contribute to the decline of erythroid and lymphoid cell populations during HSC aging. This study also found that many of the hypermethylated regions were Polycomb targets and observed diminished expression of Polycomb regulators (for example, *Ezh2*) during HSC aging, which is in agreement with our model (Figure 1). Another recent study analyzing the methylome in HSCs from 4-month old versus 24-month-old mice using whole genome bisulfite sequencing also found a small increase in DNA methylation in older stem cells (84.6 versus 83.5%) [92]. This study found that HSC-specific genes such as Gata2 and Hmga2 are hypomethylated and upregulated in old mice and that binding sites of transcription factors associated with differentiation, such as Pu.1, tend to become hypermethylated. Such changes will reinforce self-renewal of HSCs and diminish differentiation as one phenotype of aging. Furthermore, this study showed that old HSCs exhibited broader H3K4me3 peaks across HSC identity genes. An increase in H3K4me3 should be associated with reduced DNA methylation since this histone modification blocks methylation of DNA by the DNMT3A/DNMT3L complex [93].

## Conclusions

DNA methylation has become a hallmark of aging, though there is so far no proof that a change in specific DNA methylation patterns can extend lifespan [1]. Nonetheless, there is a growing body of literature that supports an age-specific drift of methylation patterns. Not only are age-dependent methylation patterns surprisingly predictive for age within a range of two to four years [17], but such signatures could also be observed in aging mice and in patients with progeroid syndrome, a disease that has many features in common with aging [94].

The age-specific drift in DNA methylation can be divided into global hypomethylation and local hypermethylation. Global hypomethylation events are enriched for repetitive sequences and thought to be responsible for the reactivation of retrotransposon elements during age, as one mechanism leading to a higher incidence of cancer. The nature of local hypermethylation changes is complex, but it has been shown that some of them co-occur near tumor suppressor genes, with possible functional effects that might be linked to cell transformation events. This is probably also true for the correlation of age-related hypermethylation and the set of Polycomb target genes, which can also be observed in various cancer types.

While certain age-specific methylation patterns can be established, the rate of age-specific DNA methylation changes does not seem to be fixed and is dependent on an array of conditions, including tissue inflammation, environmental exposures and life style influences. Especially nutrition-based decisions, including intake of essential nutrients (methionine, choline, folic acid, and vitamin B12) involved in the metabolism of methyl groups, are believed to be key factors in delaying the progressive deterioration of DNA methylation patterns. As such, the continuous maintenance of a proper one-carbon metabolism may be important for healthy aging and may slow the development of age-related pathologies [71,95].

The current challenges in age-related DNA methylation research are at least two-fold: (i) to find the precise mechanism rather than observing correlative associations and (ii) to identify the most important genes and pathways for which altered methylation patterns contribute to age-related functional decline. Our current model of *de novo* methylation during aging, at least for Polycomb-associated sites, is based on competition between DNMT3A/DNMT3B and subunits of the PcG machinery. Continuous age-related degradation of DNA-PRC1 and PRC2 complexes will increase the interaction of the target sequences with the *de novo* DNA methylation enzymes (Figure 1). In contrast to DNA mutations, epigenetic alterations are reversible and as such are promising targets for devising therapeutic approaches aimed at slowing the inevitable process of aging.
